# Energy-Balanced Routing Algorithm Based on Ant Colony Optimization for Mobile Ad Hoc Networks

**DOI:** 10.3390/s18113657

**Published:** 2018-10-28

**Authors:** Dong Yang, Hongxing Xia, Erfei Xu, Dongliang Jing, Hailin Zhang

**Affiliations:** 1 State Key Laboratory of Integrated Service Network, Xidian University, Xi’an 710071, China; dyang@mail.xidian.edu.cn (D.Y.); yhruan@xidian.edu.cn (E.X.); dongliangjing@126.com (D.J.); hlzhang@xidian.edu.cn (H.Z.); 2 Nantong Normal College, Nantong 226010, China

**Keywords:** energy constraint, ant colony optimization algorithm, convergence, remaining lifetime

## Abstract

The mobile ad hoc network (MANET) is a multi-hop, non-central network composed of mobile terminals with self-organizing features. Aiming at the problem of extra energy consumption caused by node motion in MANETs, this paper proposes an improved energy and mobility ant colony optimization (IEMACO) routing algorithm. Firstly, the algorithm accelerates the convergence speed of the routing algorithm and reduces the number of route discovery packets by introducing an *offset coefficient* of the transition probability. Then, based on the energy consumption rate, the remaining lifetime of nodes (RLT*n*) is considered. The position and velocity information predicts the remaining lifetime of the link (RLT*l*). The algorithm combines RLT*n* and RLT*l* to design the pheromone generation method, which selects the better quality path according to the transition probability to ensure continuous data transmission. As a result, the energy consumption in the network is balanced. The simulation results show that compared to the Ad Hoc on-demand multipath distance vector (AOMDV) algorithm with multipath routing and the Ant Hoc Max-Min-Path (AntHocMMP) algorithm in consideration of node energy consumption and mobility, the IEMACO algorithm can reduce the frequency of route discovery and has lower end-to-end delay as well as packet loss rate especially when nodes move, and can extend the network lifetime.

## 1. Introduction

The mobile Ad hoc network is a multi-hop temporary autonomous system formed by mobile nodes through a wireless link [1]. The nodes in MANET can dynamically join and leave the network [2]. Due to its flexible network capabilities and the lack of infrastructure, it is widely used in military and civilian applications. The typical application scenario of MANET is to establish communication between rescuers, search teams and medical personnel in the disaster area where network infrastructure is damaged [3]. In addition, it has many other applications in our daily lives, including habitat monitoring, environmental monitoring and ocean monitoring, as well as emerging application areas such as traveler positioning, target tracking and social networking [4,5,6,7,8,9].

With the use of close-range space and the rapid development of aircraft such as drones, the mobile ad hoc network scene is more diversified. However, due to the slow progress of battery technology, energy is still a bottleneck for the wider application of MANETs [10]. For energy-limited mobile Ad Hoc networks, the main challenges in the design of routing protocols are not only to ensure global convergence, but also to improve route discovery efficiency. It also needs to maximize the energy consumption of the network, ensure the continuity of data transmission, and automatically adapt to the dynamic changes of the network topology. The existing literature has conducted extensive research on energy equalization protocols. Utilizing an efficient energy approach can minimize energy consumption throughout the network and maximize network life. These protocols can generally be divided into two categories: minimum energy routing protocols and maximum network lifetime routing protocols. The minimum energy routing protocol searches for the most energy-efficient path from the source node to the destination node [11], and the maximum network lifetime routing protocol attempts to balance the residual battery power of each node when searching for energy-saving paths [12]. The ant colony algorithm has good performance in terms of energy balance and has been extensively studied.

Bionic algorithms mimic the behavior of living things on Earth. The purpose of evolution is to achieve a given goal and ultimately achieve optimal results. The approach is to encapsulate simple heuristic rules into distributed operations [13]. The ant colony optimization algorithm has many good characteristics. It is a new general heuristic algorithm framework for solving combinatorial optimization problems. It has the characteristics of positive feedback, distributed computing and dynamic adaptation. Since the ant colony optimization algorithm has the above advantages, many researchers are working in this direction [14]. The ant colony algorithm is a recently prevalent global searching algorithm owing to its perfect performance in solving optimization problems [15].

AntNet proposed in [16] is the first routing algorithm based on ant colony optimization. The local model of the network state and the local routing table are updated when the reverse ant original path returns. This provides a basic model for the application of ant colony optimization algorithms to MANET. The literature [17] designed an active routing protocol-probabilistic emergent routing algorithm (PERA). The protocol also uses ant discovery network topology and routing maintenance in dynamic networks for mobile network topologies such as MANET. The improved ant colony optimization routing algorithm for mobile ad hoc networks (PACONET) proposed in the literature [18] uses forward and backward ants to update pheromone, which is different from the AntNet algorithm. The forward ant updates the pheromone to speed up the convergence of the algorithm, but it also brings the problem of being more likely to fall into the local optimum. The forward ant overhead of the broadcast increases because the ant needs to record the total time of the pathfinding and the ID of the passed node. In order to solve the problem that the basic ant colony optimization algorithm is easy to fall into the local optimum, the literature [19] proposed the Rank-Based Version of Ant System (ASrank). The algorithm is obtained by introducing the idea of sorting in the classical genetic algorithm into the ant system.

The literature [20] designed an ant-swarm inspired energy-efficient ad hoc on-demand routing protocol (ACO-EEAODR) by considering the residual energy and path length of the node. The weighting factors of the two parameters in the protocol take different values according to different scenarios, and the pheromone of each node is updated according to the residual energy. Therefore, in this protocol, the selection of path is more inclined to nodes with more node energy than the shortest path, and the network energy consumption is balanced to some extent. In [21], Ant System concept is used in the mesh routing networks in order to discover the best routes while maintaining the distributed coordination between the nodes in Low-Power and Lossy network(LNNs) with low power consumption. The Ant Colony Optimization in [21] we called LNNACO improves packet transmission rate by considering energy consumption in the MMP ant colony algorithm. Because the LNNACO algorithm does not consider the impact of high mobility of nodes, it is not suitable for networks with higher mobile speeds. The literature [22] proposed an energy-aware ant-based routing (EAAR). The algorithm considers the node energy consumption based on the multipath routing protocol. In the route discovery phase, the maximum minimum residual energy (MRB) and route hop count on the path are used to update the pheromone. However, the packet delivery rate and the energy per packet in high mobile conditions are relatively inferior for EAAR. This is because energy-awareness increases the time to judge the best route for transmission. Unlike EAAR, which considers only residual energy, the literature [23] proposed an ant colony-based energy control routing protocol (ACECR). The ACECR considers the average residual energy and the minimum residual energy for each path. In the route discovery phase, the backward ant updates the pheromone table based on the minimum energy, average energy, and hop count in the statistics. However, ACECR does not guarantee that the total transmission power is minimized over a chosen route. The literature [24] used the simulated annealing algorithm (SA) to find the global optimal path, combining SA with ACO. The algorithm first selects the next hop node with SA, and then updates the pheromone with the rule of ACO. The downside existing in this approach is that the time taken for determining the routing path and the distance of the routing path remains high when compared to the regular ACO algorithm. In the literature [25], by combining the firefly algorithm with ACO, the firefly algorithm is used to obtain the list of the leader nodes, the distance between the nodes, and the density of the nodes. The feature of the firefly algorithm is that the optional set of the next hop is the area with a large density of nodes. The convergence speed is faster, and then the ACO’s transition probability strategy is used for routing. On the basis of the literature [26], the value of the equilibrium constant is adjusted according to the number of iterations. At the beginning of the iteration, the ant explores the new path and discovers the path to the destination node in the network topology as much as possible. After a period of time, the topology discovery in the network tends to be stable, the equilibrium constant is reduced, and the convergence speed of route discovery is accelerated. However, the algorithm ignores the fact that the pheromone concentration of some paths is significantly higher than that of other paths, which leads to more iterations to find the optimal path and reduces the convergence speed of the algorithm.

In summary, the literature [17,24,25] just improved the convergence speed of the algorithm, while the literatures [20,22,23] only improved the energy aspect of the node. Aiming at these problems, in this paper, from the perspective of topology-based routing protocols, considering the mobility and energy-constrained features of nodes, the ant colony optimization algorithm is used to design routing protocols. This paper proposes an improved algorithm IEMACO which comprehensively considers the convergence of the ant colony algorithm and node energy balance. Firstly, the algorithm judges the offset of the transition probability based on random numbers. This improvement accelerates the convergence speed of the algorithm on the basis of ensuring global optimization. Then, this paper uses the remaining lifetime based method to define the robustness of the link, thereby improving the Key Performance Indicators (KPIs): the end-to-end delay and the Packet Delivery Ratio [27] and other network performance.

## 2. Preliminaries

Ant Colony Optimization, proposed by Italian scholar Marco Dorigo et al. [28], is a heuristic evolutionary algorithm based on bionics with the characteristics of distributed computing, simple implementation, good robustness and self-adaptability [29]. ACO finds the optimal path from the source node to the destination node through multiple iterations with positive feedback mechanism. The characteristics of self-organization, randomness, dynamic and distributed make the ant colony optimization algorithm very similar to an ad hoc network. At present, a large number of scholars have applied ant colony optimization algorithm to ad hoc networks.

### 2.1. Principle of the Ant Colony Optimization Algorithm

In an ant colony optimization algorithm, the choice of each path depends on two aspects: pheromone concentration and heuristic information. Pheromone refers to the chemical substances secreted by each ant in the process of finding a path. When the ant successfully finds the target node, pheromones will increase in a certain proportion. Heuristic information is a priori knowledge about the link, and, in most cases, it refers to the cost of selecting the path or the connection state of the link. The ant colony optimization algorithm is mainly divided into two parts: the transition probability criterion and the parameter definition. The criterion of transition probability is how to choose the next hop according to the transition probability. The basic expression of transition probability is as follows:

(1) $$p_{ij}\left(t\right)=\left\{\begin{array}{cc} \frac{\tau_{ij}^{\alpha }\left(t\right)g\eta_{ij}^{\alpha }\left(t\right)}{\sum_{l\in N_{i}}\tau_{il}^{\alpha }\left(t\right)g\eta_{il}^{\beta }\left(t\right)}, & \mathrm{if}\;j\in N_{i},\\ 0, & \mathrm{if}\;j\notin N_{i}, \end{array}\right.$$

where $p_{ij}\left(t\right)$ is the probability that the ant moves from node *i* to node *j* at time *t*; $N_{i}$ is the set of optional next hop nodes of node *i*; $\tau_{ij}\left(t\right)$ represents the pheromone concentration on the link at time *t*; $\eta_{ij}\left(t\right)$ denotes the heuristic information on the link at time *t*, which usually refers to the connection status of the link or the cost of selecting the path. α is the pheromone heuristic factor, indicating the role played by the pheromone released by the ant in the path selection. The greater its value is, the stronger the indirect effect between ants will be. In other words, the path taken by the forwarded data packet has a greater influence on the selection of the current path, then eventually all ants select the same path. If α=0, it degrades into the greedy algorithm (local optimum selection), and the ant selects only the path which is currently considered best. β is the expected heuristic factor, indicating the relative importance of heuristic information. The higher its value is, the greater the influence of link quality on the packet selection path will be. In the transfer probability formula, the emphasis is on pheromone and heuristic information. Other weight factors can be set according to different scenarios by using empirical values or simulation analysis. When an ant completes a routing or selects the next hop, or the update cycle set by the routing protocol comes, the pheromone of the selected path is updated according to Formula (2):

(2) $$\tau_{ij}\left(t+1\right)=\left(1-\rho \right)\tau_{ij}\left(t\right)+\rho \Delta \tau_{ij}\left(t\right),$$

where $\Delta \tau_{i,j}\left(t\right)$ is the amount of pheromone that the ant deposits this time on the link (i,j); ρ is the pheromone evaporation rate, indicating the rate that the pheromone decays over time, and the value range of ρ is ρ∈[0,1], so 1−ρ indicates the residual level of the pheromone. In different algorithms, the expressions of $\Delta \tau_{i,j}\left(t\right)$ or $p_{i,j}\left(t\right)$ are distinct depending on specific problems.

### 2.2. AntHocMMP Algorithm

Due to the dynamic topology of the MANET network, the link is easy to disconnect. Therefore, it is necessary to design reliable and adaptable routing protocols. In Reference [30], an improved ant colony optimization algorithm named AntHocMMP is proposed, which combines three methods to obtain higher packet delivery rate, prolong the survival time of the network, and reduce the number of re-transmissions, thus reducing the end-to-end delay. The first method considers the residual energy and energy consumption of the node, and calculates the quality of the path by using the maximum minimum path algorithm; the second method updates the pheromone by the forward ant and considers the energy consumption of the path. Periodic pheromone updates can improve the adaptability of the algorithm to dynamic topologies. The third method is to predict the link failure time and use the maximum minimum path algorithm to find the alternate path. In order to detect the link failure in time, each node broadcasts a hello packet to the neighbor node. If a link failure is detected, the alternative path is selected using the transition probability criterion, and the transfer rule [30] is as shown in formula (3):

(3) $$p_{ij}^{}\left(t\right)=\left\{\begin{array}{cc} \frac{\tau_{ij}^{\alpha }\left(t\right)}{\sum_{d\in N_{i}}\tau_{id}^{\alpha }\left(t\right)}, & \mathrm{if}\;j\in N_{i},\\ 0, & \mathrm{else}. \end{array}\right.$$

After the ants arrive at the destination node, reverse ants are immediately generated and return along the original path. The pheromone table in each node is updated at the same time.

## 3. IEMACO Routing Algorithm

The AntHocMMP routing algorithm can achieve higher packet delivery rates and lower end-to-end delays by combining the three methods. However, in the AntHocMMP routing algorithm flow, the convergence speed of the algorithm and the influence of energy consumption on the generation and update of the pheromone are not considered. In order to speed up the route discovery and prolong the network survival time, this paper proposes an IEMACO routing algorithm, which is designed from three aspects: algorithm convergence, pheromone generation and pheromone update.

### 3.1. Algorithm Convergence

In the ant colony algorithm, after the ant completes a path search, the pheromone will be accumulated along the way thus the next ant will prefer the found route. In other words, the ant colony optimization algorithm has positive feedback characteristics, which will cause falling into local optimum. In Reference [26], the routing criteria of the ant colony optimization algorithm are improved as follows: the balance coefficient $q_{0}$ is introduced to balance the importance of the ant to explore new paths and to utilize existing information. When $q>q_{0}$, the ant uses the existing information to route, that is, uses the transfer probability formula to select the route; when $q\leq q_{0}$, the ant explores a new path, and randomly selects one node from the set of reachable nodes as the next hop. The corresponding transition probability is formulated as the following:

(4) $$p_{ij}\left(t\right)=\left\{\begin{array}{ccc} \frac{\tau_{ij}^{\alpha }\left(t\right)\cdot \eta_{ij}^{\beta }\left(t\right)}{\sum_{l\in N_{i}}\tau_{il}^{\alpha }\left(t\right)\cdot \eta_{il}^{\beta }\left(t\right)}, & \mathrm{if}\;j\in N_{i}\;\&\&\;q>q_{0} & \;(a),\\ 1/N, & \mathrm{if}\;j\in N_{i}\;\&\&\;q<q_{0} & \;(b),\\ 0, & \mathrm{if}\;j\notin N_{i} & \;(c). \end{array}\right.$$

Here, the random number *q* satisfies q∈(0,1), *N* indicates the number of reachable nodes of the node *i*. The smaller the $q_{0},$ the more likely it is that the ant is selected according to the current pheromone concentration. The greater the $q_{0},$ the more likely the ant is to explore the new path. For the single-path routing algorithm, the path with the highest transition probability is selected when case (a) in Equation (4) is satisfied. When case (b) in Equation (4) is satisfied, the random routing is performed. In this case, since it ignores the fact that the pheromone concentration of some paths is significantly higher than that of other paths, it requires more iterations to find the optimal path, which hinders the convergence of the algorithm and the speed of route discovery. The designing of adjusting in state transition optimization rules are as follows: choose pheromone larger side when algorithm starts running. In order to prevent falling into local optimal solution, change the value of $q_{0}$ and design a random function to generate a random number making the algorithm converges to the global optimal solution.

To speed up the convergence, in this paper, we introduce a new parameter θ, named *offset coefficient* which is the deviation to the mean pheromone concentration, to split case (b) in Equation (4) into two sub-cases. The key idea is that, when the transition probability (calculated by formula (a)) of a certain path is significantly larger than that of other paths, directly selecting it as the next hop can accelerate the convergence speed of the algorithm. The proposed transition probability then can be formulated as follows:

(5) $$p_{ij}\left(t\right)=\left\{\begin{array}{ccc} \frac{\tau_{ij}^{\alpha }\left(t\right)\cdot \eta_{ij}^{\beta }\left(t\right)}{\sum_{l\in N_{i}}\tau_{il}^{\alpha }\left(t\right)\cdot \eta_{il}^{\beta }\left(t\right)}, & \mathrm{if}\;j\in N_{i}\;\&\&\;q>q_{0} & \;(a),\\ \max(p_{ij}), & \mathrm{if}\;j\in N_{i}\;\&\&\;q\leq q_{0},\;\exists j\;p_{ij}>\theta /N & \;(b1),\\ 1/N, & \mathrm{if}\;j\in N_{i}\;\&\&\;q\leq q_{0},\;\forall j\;p_{ij}\leq \theta /N & \;(b2),\\ 0, & \mathrm{if}\;j\notin N_{i} & \;(c). \end{array}\right.$$

When θ≥N, $P(p_{ij}>\theta /N)=0$, the routing criteria degenerate to the random number routing criteria as in Reference [26]. When θ=0, $P(p_{ij}>\theta /N)=1$, the routing criteria for the case $q\leq q_{0}$ is directly selecting the node with the highest transition probability as the next hop. Therefore, the selection of θ is important, it must ensure the global optimization of the algorithm, and accelerate the convergence speed of the algorithm as much as possible. In this paper, the appropriate θ is selected by comparing simulation results with varying parameters. The proposed IEMACO considers the case of $q\leq q_{0}$, when each node in the candidate set corresponds to its own transition probability. When the transition probability of a certain path is significantly larger than other paths, it is directly selected as the next hop can speed up the convergence of the algorithm while ensuring the global convergence of the algorithm.

### 3.2. Generation of Pheromone

Energy is the bottleneck in mobile Ad Hoc networks. Once the node is exhausted, its path is immediately invalid, resulting in data transmission failure and higher end-to-end delay. This paper introduces the path minimum residual energy, average energy and energy consumption rate into the initialization of each node pheromone in the path. The remaining lifetime of nodes (RLT*n*) and the remaining lifetime of links (RLT*l*) are defined based on the prediction of the state of the network, while the remaining energy and the average energy are based on the metrics of the current network state.

#### 3.2.1. Remaining Lifetime of Nodes

The energy consumption model adopted in this paper is similar to Reference [29]. It is assumed that node *S* sends *k* bits data to node *D* with distance *d*. Node energy is mainly spent on transmitting and receiving of data. When one packet is transferred from node *i* to next hop node *j*, the energy consumed is c(i,j),

(6) $$c\left(i,j\right)=2E_{elec}\cdot k+E_{0}\cdot k\cdot d^{2},$$

where $E_{elec}$ indicates the energy consumption for processing one bit data, $E_{0}$ represents the energy consumed by the amplifier to send a one-bit data. The energy consumed on each path is c(S,D),

(7) $$c\left(S,D\right)=\sum_{i=S}^{D-1}c(i,i+1).$$

The energy of the node is constantly being consumed as the data is transmitted. The energy of a node at any time can be expressed as

(8) $$E_{i}\left(t\right)=E_{i}\left(t\right)-c\left(i,j\right).$$

Here, we introduce the definition of the remaining lifetime of node *i* as

(9) $$\mathrm{RLT}n_{i}=\frac{E_{i}}{r_{i}},$$

where $r_{i}$ represents the energy consumed by the node per unit time, i.e., the energy consumption rate [31],

(10) $$r_{i}\left(t\right)=\frac{E_{i}\left(t+\Delta t\right)-E_{i}\left(t\right)}{\Delta t}.$$

#### 3.2.2. Remaining Lifetime of Links

The calculation method of the remaining lifetime of links in this paper is as follows: assuming that the moving speed, moving direction and geographical position of each node of the network are known, the remaining lifetime of the link between node *i* and node *j* can be calculated as

(11) $$\mathrm{RLT}l_{ij}=\frac{-\left(ab+cd\right)+\sqrt{\left(a^{2}+c^{2}\right)r^{2}-{\left(ad-cb\right)}^{2}}}{a^{2}+c^{2}},$$

where $a=v_{i}\cos\theta_{i}-v_{j}\cos\theta_{j}$, $b=x_{i}-x_{j}$, $c=v_{i}\sin\theta_{i}-v_{j}\sin\theta_{j}$, $d=y_{i}-y_{j}$. *r* represents the maximum communication distance of nodes in the network. A longer remaining lifetime indicates a more stable link. In the dynamic network, it requires a stable link to avoid data retransmission, improve transmission efficiency, save energy consumption and reduce route discovery frequency.

#### 3.2.3. Generation of Pheromone

The remaining lifetime of nodes and links proposed in this section are based on predictions of network status, while residual energy and average energy are assessments of the current status of the network. The pheromone initialization indicates the quality of each path obtained by the method of randomly selecting the *forward ants* and updating the state to the *backward ants* after the end of the route discovery phase. The pheromone is generated by the following equation:

(12) $$\tau_{0}=\frac{E_{min}\cdot E_{avg}}{L_{path}}\cdot T,$$

where $E_{min}$ represents the minimum residual energy of all nodes to be accessed on the path, $E_{min}=\min E_{i}\left(i\in path\right)$. *T* represents the minimum remaining lifetime of all nodes and links to be accessed on the path, T=min(min(RLTn),min(RLTl)). $E_{avg}$ is defined as the total energy remaining in the path to be accessed divided by the path length, $E_{avg}=\sum_{i\in path}E_{i}/L_{path}$. The path length $L_{path}$ is defined as the sum of the length of each hop to be accessed.

### 3.3. Update of Pheromone

The update of the pheromone means that, after each ant successfully finds the destination node or the data is transmitted on the path, the pheromone is deposited on the path. As time goes by, the pheromone will periodically evaporate, which helps to find the optimal path and accelerate the convergence of the algorithm. The residual energy and the distance of the node are considered in the update of the pheromone, and the reward and punishment mechanism is introduced [26]. When the forward ant finds the path successfully, the backward ant generated by the destination node returns along the original path and updates the pheromone (reward). When the ant fails to find a path, the last node generates a backward ant to return along the original path and update the pheromone (punishment). The update rules are as follows:

(13) τ(t+1)=(1−ρ)·τ(t)+ρ·Δτ,

and

(14) $$\Delta \tau =I_{RS}\cdot \frac{E_{min}}{L_{path}},$$

where $I_{RS}$ indicates the routing result. If the ant routes successfully, $I_{RS}=1$; otherwise, $I_{RS}=0$. If a path does not pass ants for a long time, on the one hand, it indicates that the path may have a low pheromone, and, on the other hand, the path may have failed. Therefore, the pheromone needs to be evaporated periodically.

(15) $$\tau \left(t+t_{0}\right)=\rho \cdot \tau \left(t\right).$$

Here, $t_{0}$ is the evaporation period. Whenever the node completes the data transmission, it triggers the timer and starts to evaporate the pheromone. All the symbols used in this algorithm are described in Table 1.

### 3.4. Protocol Design

The routing protocol designed in this paper is based on the AntHocMMP routing protocol. It improves the broadcast phase and the returning process for the *backward ant*. The route discovery process is as follows.

**Step 1**. After the network is initialized, when the source node needs to transmit data to the destination node, query whether there is a path to the destination node in the local routing table. If so, enter the route maintenance (data transmission phase). If the source node does not have any routing information of the destination node, the source node generates a *forward ant* Fant and broadcasts.

**Step 2**. When the node receives the Fant packet, check whether it has been forwarded by itself or generated by itself. If yes, discard it directly. Otherwise, check whether the lifetime has expired. If yes, discard it directly. Otherwise, check whether the destination node ID is its own ID, if not, write its own ID to the path, modify the hop count and broadcast. If the node is the destination node, go to step 3.

**Step 3**. When the broadcast packet arrives at the destination node, first check the node information in the path, generate a *backward ant* Bant, then return it according to the node information of the path domain. In addition, when the destination node receives the first forward ant, it starts a timer, and the Fant that arrives at the destination node within the specified time will be received. Otherwise, the route discovery time is too long and the link quality is poor.

**Step 4**. Establish a node state table and a pheromone-transfer probability table according to the node information in the Bant path state table and previous formulas.

**Step 5**. After the Bant reaches the source node, the pheromone table and the state transition table are established, and the route discovery phase ends.

**Figure 1:**
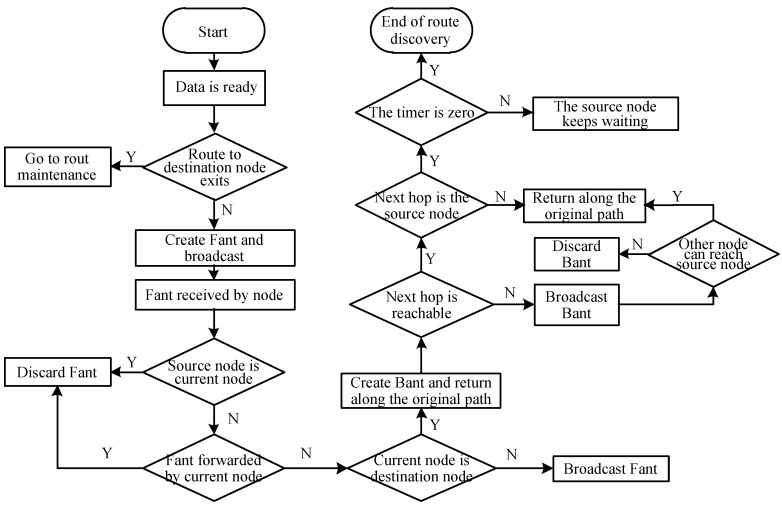
Flow of the route discovery.

The detailed route discovery process is shown in Figure 1. The pseudocode of the IEMACO routing algorithm is provided in Algorithm 1.

For simplicity, the corresponding expressions are shortened as follows: phr-tab for pheromone table, st-rt-tab for stack routing table, $N_{dest}$ for destination node, $N_{src}$ for source node; $P_{optimal}$ for optimal path, $P_{robust}$ for robust path, $N_{current}$ for current node, $N_{neigh}$ for neighbor node and link-disconn for link disconnection, ep for energy path costs, used to update the phr-tab when initializing the network.

**Algorithm 1** IEMACO protocol
**Input:** phr-tab, $N_{dest}$, $N_{src}$, $N_{current}$, $N_{neigh}$
**Output:** $P_{optimal}$
 Initialize the network by using Equations (9) and (11) **if** There is a path $P_{local}$ to $N_{src}$ **then return** $P_{optimal}=P_{local}$ **else** $P_{robust}$ = generation of FANT by $N_{src}$ **end if** **if** $P_{robust}\neq 0$ **then**   Update pheromones in phr-tab by using Equation (12)   Update phr-tab = phr-tab ($N_{current}$, $N_{neigh}$)   st-rt-tab ← st-rt-tab($N_{current}$) **end if** **if** FANT = $N_{dest}$ **then**   Generation of BANT by $N_{dest}$   Update phr-tab and return $P_{robust}$ **end if** **if** Next hop is not reachable **then**   link-disconn =detection and maintenance of link   disconnections during traversal of the ants **end if** **if** link-disconn= 0 **then**   Select $P_{optimal}$ between $N_{src}$ and $N_{dest}$   $P_{optimal}=P_{robust}$ **else**   Restart the generation of FANT by $N_{src}$ **end if**   **return** $P_{optimal}$


We next discuss the space and time complexity of the IEMACO algorithm. Without loss of generality, we assume the number of nodes in the network is *n* and the number of ants in each iteration is *m*. In Reference [32], the space complexity of the basic ant colony algorithm has been analyzed and is proved to be O(n∗n)+O(n∗m). For the improved ant colony algorithms, when the scale of the problem is the same, its space complexity is similar to that of basic ACO. For AntHocMMP and IEMACO, more two-dimensional matrices are needed to store the energy cost than the basic ACO. However, the space complexity of AntHocMMP and IEMACO is the same as that of basic ACO, which is O(n∗n)+O(n∗m).

The time complexity of each iteration has been analyzed in literature [33]. For AntHocMMp and IEMACO, the time complexity of each step in the algorithm implementation process can be obtained by Table 2. If the algorithm circulates $N_{c}$ times, the time complexity of the AntHocMMP algorithm is $O(N_{c}*n^{2}*m)$ which is same with the basic ACO. Since the IEMACO algorithm introduces the acceleration convergence mechanism, the time complexity is reduced to $O(log\left(N_{c}\right)*n^{2}*m)$.

## 4. Simulations

In this section, we first use MATLAB R2016b to simulate and verify the convergence of the improved ant colony optimization algorithm. Then, the IEMACO protocol designed in this paper is simulated by OPNET. The simulation scenario is set to a rectangular area of 100 m × 100 m. The source node coordinates are (0, 0) and the destination node coordinates are (100, 100). Other 40 nodes are uniformly distributed within the rectangular.

### 4.1. Verification of Algorithm Convergence

We assume that the source node periodically broadcasts ants for route discovery, and each ant selects the route according to the transition probability Equation (5). It is assumed that all nodes have the same communication range, all nodes initially have the same energy, and the energy consumption is mainly determined by the distance between the nodes. The simulation parameter settings are shown in Table 3. These parameters in the table were defined empirically [21].

The offset coefficient θ has a range of [0,N], in our simulations θ takes 0,0.1N,⋯,0.9N. At the beginning of the simulation, the pheromone on all nodes is initialized first, and 10 ants are released for random routing in each iteration. When the node reaches the destination node (100,100), the pheromone of all nodes on the corresponding path is updated immediately. After *n* iterations, the average length of the path selected by the ant is obtained.

**Figure 2:**
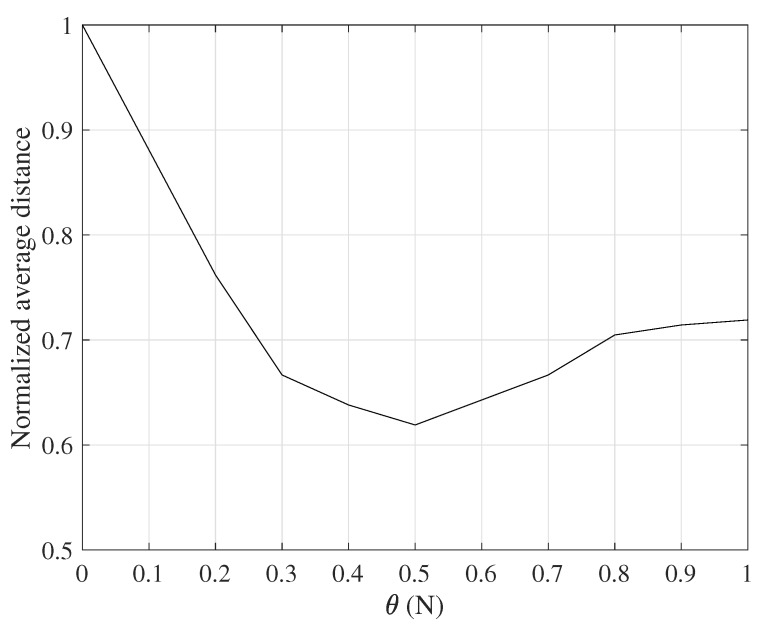
Routing performance with varying *offset coefficient* θ.

The normalized average distance in Figure 2 refers to the average distance after iterating *n* times divided by the farthest distance from the source node to the destination node in the route discovery process. It can be seen from Figure 2 that, when θ=0.5N or $p_{ij}>0.5$, the path obtained by the same number of iterations is the shortest; when θ=0, that is, $p_{ij}>0$, the obtained path length is the longest. This is because the forward ant directly selects the path with the highest probability as the next hop for each iteration, and falls into the local optimum, and the difficulty of finding the new path by the ant is increased due to the accumulation of pheromone. When θ≥N, or equivalently $p_{ij}>1$, the obtained path is also not optimal. This is because the routing criterion degenerates into random routing, and more iterations are needed to find the optimal path.

**Figure 3:**
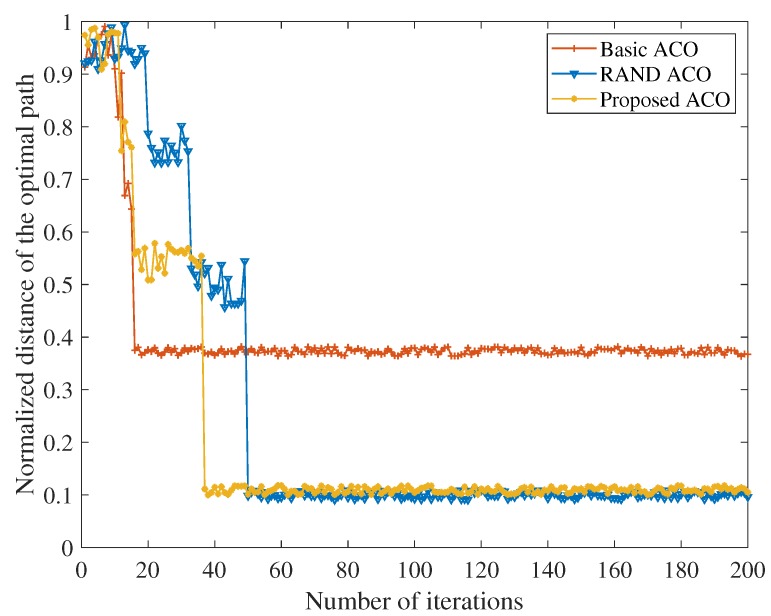
Comparison of convergence performance.

Based on the optimal θ, in Figure 3, the improved ant colony optimization algorithm, basic ant colony optimization algorithm and random number based ant colony optimization algorithm are simulated and compared. It can be seen from Figure 3 that the convergence performance of the improved ant colony optimization algorithm proposed in this paper is basically consistent with the ant colony optimization algorithm that introduces random numbers. The global convergence of the ant colony optimization algorithm is thus verified. In addition, the path obtained by the basic ant colony optimization algorithm is significantly worse than the other two methods. This is because the positive feedback mechanism of the ant colony optimization algorithm makes the pheromone accumulation speed too fast, which makes it difficult to find new paths in subsequent iterations, and makes the algorithm fall into local optimum. At the convergence rate, the basic ant colony optimization algorithm can be seen to be the fastest because the criterion for path routing is the pheromone concentration. Compared with the algorithm which introduces random numbers, the algorithm proposed in this paper introduces the transition probability, so the number of iterations is significantly reduced, and the convergence speed is obviously improved. The IEMACO improved in this paper can obtain the convergence speed higher than the ACO with random numbers, but achieves the optimal path similar to the basic ACO.

### 4.2. Performance of Routing Protocol

In this section, the IEMACO routing protocol proposed in this paper is simulated and analyzed with an OPNET network simulation tool. The comparison routing protocols are AOMDV and AntHocMMP. We also compare part of the performance of IEMACO with LNNACO. To evaluate the performance of routing protocols, the end-to-end delay, packet delivery rate and route discovery frequency are considered. In addition, since the energy consumption rate of the nodes is considered in this paper, the death time of the first node and the number of dead nodes at the end of the simulation are compared to measure the energy balance performance of the routing protocol. The simulation parameters are shown in Table 4.

**Figure 4:**
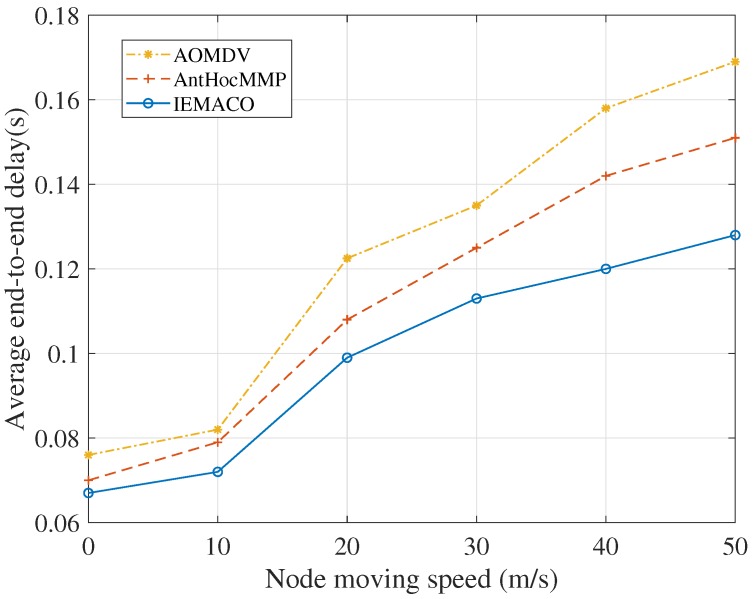
Average end-to-end delay versus node moving speed.

**Performance comparison with different node speeds**. It can be seen from Figure 4 that, with the increase of node moving speed, the average end-to-end delay in the network gradually increases. The IEMACO protocol designed in this paper has the lowest delay at different moving speeds, while the AOMDV has the largest delay. This is because the routing design of AOMDV does not consider the mobility of the node. When the link is disconnected, it takes longer to discover a new path. AntHocMMP adopts multipath based on the energy of the node, and uses the two parameters of rate and delay in routing, avoiding the path with large rate and large delay. IEMACO considers the distance and speed between nodes and selects the path with longer remaining lifetime, so the end-to-end delay is minimal.

**Figure 5:**
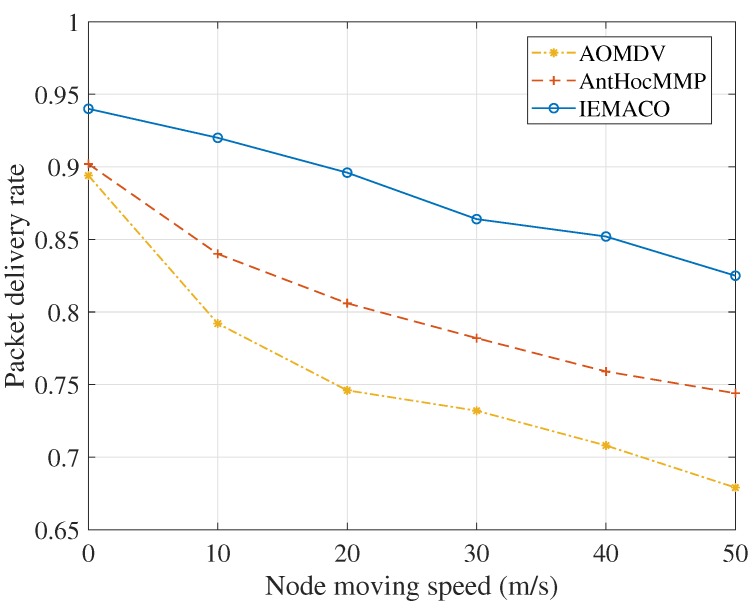
Packet delivery rate versus node moving speed.

As can be seen from Figure 5, as the node moves faster, the packet delivery rate decreases. This is because the node movement causes the data packet being transmitted to be unable to find the next hop, and the lifetime is exhausted; then, the packet is discarded. When the node is static, the packet delivery rate is not 1 because, in each simulation, there is always a node whose energy is exhausted over time, resulting in partial packet loss. However, because IEMACO considers more about energy balance of the network than AntHocMMP and AOMDV, the node failure occurs less and the packet delivery rate is higher.

**Figure 6:**
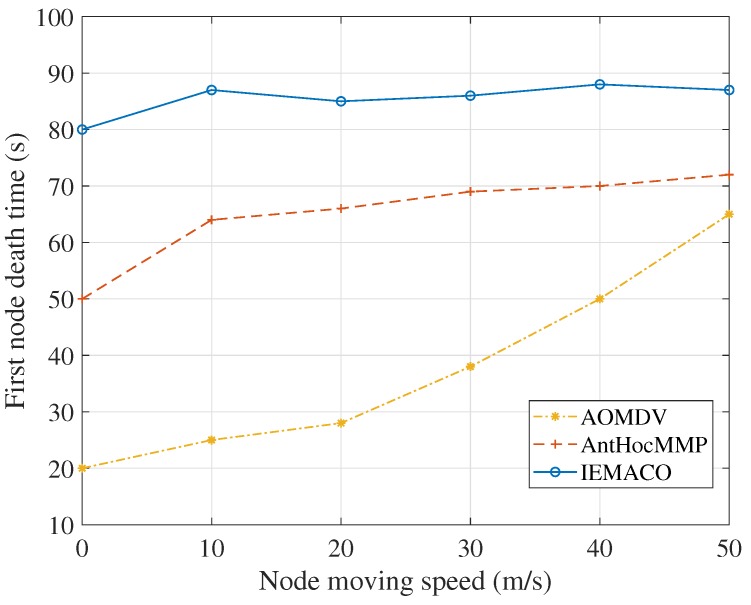
First node death time versus node moving speed.

Figure 6 shows the death time of the first node at different node movement speeds. As can be seen from Figure 6, for the two routing protocols compared in this paper, when the node moving speed increases, the dead time of the first node increases. It can be explained as that each node passively handles the topology change of the network and the transmission of data is spread over different nodes; therefore, the energy consumption of a single node is relieved to some extent. The protocol proposed in this paper actively detects the link state when routing and avoids the path with fast moving speed and high energy consumption, so it can balance energy consumption. At the same time, IEMACO’s first node death time did not increase with the increase of node speed but remained at about 85 s. This is also because the network topology change and node energy state are considered in routing.

**Figure 7:**
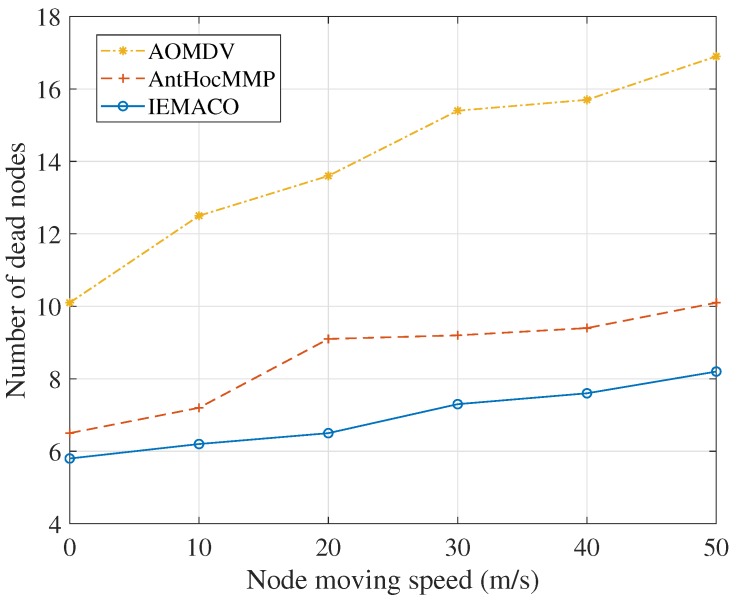
Number of dead nodes versus node moving speed.

Figure 7 shows the relationship between the number of dead nodes and node moving speeds. It can be seen that the number of deaths of the node increases as the moving speed of the node increases. This is because the data retransmission and the number of route discovery times increase, so that the energy consumption of the node increases. Compared to IEMACO, the number of dead nodes in AOMDV increases significantly due to measuring the link quality by distance. On the contrary, IEMACO selects the path with better link quality according to the residual energy, energy consumption rate and node speed, so that it can adapt to network topology changes and balance energy consumption. Therefore, the number of dead nodes is not significantly affected by the speed of the node.

In summary, as IEMACO introduces the remaining life time of the link in routing by considering the node speed direction and the node distance, it can avoid selecting the path that is easy to break, reduce the route discovery frequency and increase the first node death time. Therefore, the average end-to-end delay of the network is reduced, the packet delivery rate is improved, and the performance of the network is improved as a whole.

**Figure 8:**
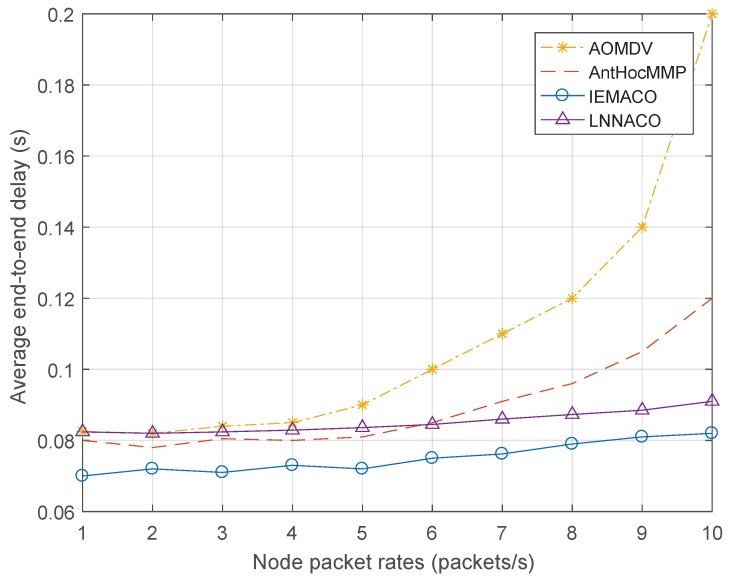
Average end-to-end delay versus node packet rate.

**Performance comparison with different network load**. Figure 8 shows the average end-to-end delay at different node packet rates. It can be seen that, as the node packet rate increases, the average end-to-end delay increases in varying degrees. For AOMDV, the data is transmitted through the main path while the processing capacity of each node is limited, once the packet transmission rate increases, the cache queue of the node increases. For this reason, the end-to-end delay of AOMDV grows fast. AntHocMMP and IEMACO route through multipath, the load increase is spread over different paths, so the delay increases slowly. IEMACO achieves the lowest end-to-end delay because the last hop node performs route discovery when the next hop node is unreachable when the link fails, which can significantly reduce the end-to-end delay. Since the probability of a route to be selected is calculated based on the sum of pheromone in the literature [21], the operation becomes simple, but the convergence speed is slow. In addition, because the energy metric takes into account the values of processing, transmission and reception, the curve changes less.

**Figure 9:**
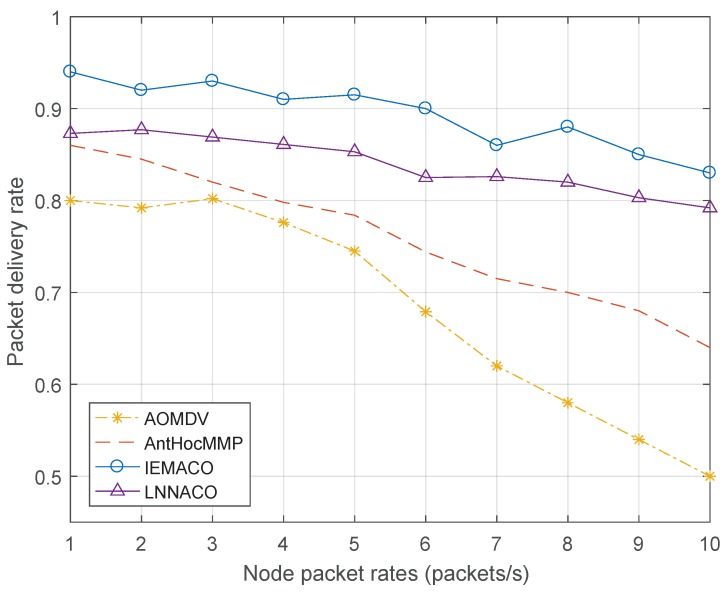
Packet delivery rate versus node packet rate.

Figure 9 shows the change in packet delivery rate at different node packet rates. As the packet sending rate increases, the packet delivery rate decreases in varying degrees. The reason is that, as the network load increases, the node is limited by the processing capability, causing network congestion, so the packet loss rate increases, and the packet delivery rate decreases. Compared with the other two routing protocols, the performance of IEMACO proposed in this paper is obviously improved. IEMACO combines the speed and the distance of the node to actively avoid selecting the nodes with higher speed. In addition, by calculating the energy consumption rate of the nodes, the load of the network is dispersed to different paths, and the nodes with longer remaining lifetime tend to be more possible to be selected. LNNACO does not consider the impact of node movement, so it will cause packet loss due to link breakage caused by node movement. Similarly, because the energy metric takes into account the values of processing, transmission and reception, the curve changes less.

**Figure 10:**
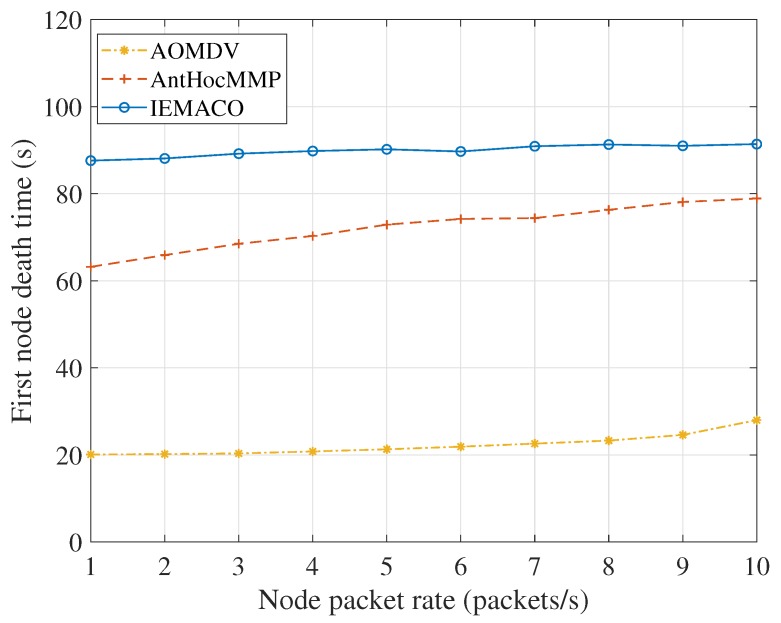
First node death time versus node packet rate.

Figure 10 compares the first node death time at different node packet rates. It can be seen from the figure that the first node dead time of the node decreases as the packet sending rate increases. The reason is that the increase in load means an increase in energy consumption, but, because the nodes are constantly moving, the intermediate nodes are constantly changing, so the energy consumption is relatively balanced, and the death time of the first node does not change much. In addition, since IEMACO considers the energy consumption rate of the node and the remaining life time of the link in the path selection, it can actively avoid selecting nodes with fast energy consumption rate and fast node movement speed, and the efficiency of network load balance is higher than that of AntHocMMP.

**Figure 11:**
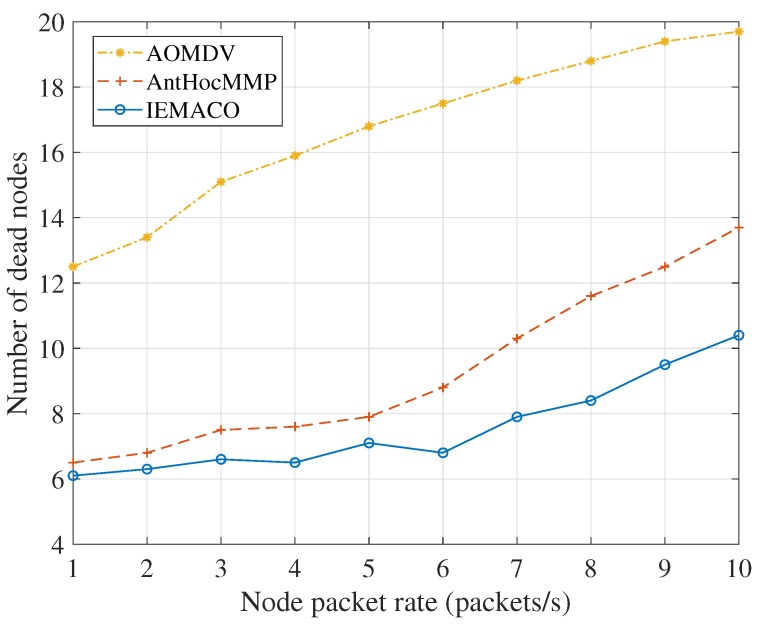
Number of dead nodes versus node packet rate.

Figure 11 indicates a simulation performance of the number of dead nodes as the node’s packet rate changes. As the load increases, the number of dead nodes increases continuously because the energy consumption accumulates continuously regardless of the primary path or multipath. As the simulation time increases, the node death is inevitable. When transmitting data, AOMDV first selects the primary path for transmission. Only when the primary path is unavailable is the backup path enabled. Therefore, the network load is mainly concentrated on one path, causing the node failure to occur frequently. AntHocMMP takes into account the residual energy of the node and the average energy of the link when routing, thus nodes with more energy will transmit more packets. In short, as the network load increases, the energy consumption of the node increases resulting in link failure. The data retransmission will bring longer end-to-end delay and packet loss rate. IEMACO considers the energy consumption rate of the node. In path selection, the nodes with less residual energy and heavy load will not be used, which can balance the network energy and prolong the service time of the network.

## 5. Conclusions

In order to accelerate the convergence speed of ant colony optimization algorithm in MANET routing, the IEMACO algorithm is proposed to select the next hop according to the transition probability *offset coefficient*. On this basis, this paper defines the pheromone generation method based on the remaining lifetime of the node and the remaining lifetime of the link, which combines node distance and residual energy for pheromone update. The simulation results show that the improved ant colony optimization algorithm can speed up the convergence of the algorithm under the constraint of global convergence, making it more suitable for mobile Ad Hoc networks with changing network topology. Furthermore, the suggested protocol for IEMACO can balance the network load and prolong the survival time of the network. At the same time, when the network topology changes, it can sense and select the path with better link quality in advance, reduce the route discovery frequency, and improve the overall performance of the network. In the future study, we will consider the algorithm performance under different parameter settings in the method of overcoming local optimal defects. 

## Figures and Tables

**Table 1:** Notation table.

Notation	Description	Notation	Description
p(i,j)	The transition probability from node *i* to node *j*	RLT*l*	The remaining lifetime of links
α	The pheromone heuristic factor	c(i,j)	The energy consumed in one hop when one packet is transferred
β	The expected heuristic factor	$E_{elec}$	The energy consumption for processing one bit data
ρ	The pheromone evaporation rate	$E_{0}$	The energy consumed by the amplifier to send a one-bit data
θ	The offset coefficient	$E_{i}$	The energy of a node at any time
*q*	A random number in (0,1)	$r_{i}$	The energy consumed by the node per unit time
$q_{0}$	The balance coefficient	$E_{min}$	The minimum residual energy of all nodes to be accessed on the path
$t_{0}$	The evaporation period	$E_{avg}$	The total energy remaining in the path to be accessed divided by the path length
RLT*n*	The remaining lifetime of nodes	$l_{path}$	The sum of the length of each hop to be accessed

**Table 2:** Time complexity.

Step	AntHocMMP	IEMACO
Initialize the network	O(n∗n+m)	O(2∗n∗n+m)
Generation of FANT	O(m)	O(m)
Update pheromone	O(n∗n)	O(2∗n∗n)
Generation of BANT and the amount of track updates	O(n∗n∗m+n∗m)	O(n∗n∗m+n∗m)
Determine if $P_{optimal}$ is appropriate	O(m∗n)	O(m∗n)
return $P_{optimal}$	O(1)	O(1)

Note: FANT, BANT and $P_{optimal}$ represnt *forward ant, backward ant* and the optimal path, respectively.

**Table 3:** Simulation parameters for ant colony optimization algorithm.

Parameter	Value	Parameter	Value
Simulation times	10,000	Scenario size	100 m × 100 m
Number of iterations	100	Ant number in each iteration	10
Pheromone heuristic factor α	0.7	Expected heuristic factor β	0.3
Initial pheromone $\tau_{0}$	10	Pheromone evaporate rate	0.7

**Table 4:** Simulation parameters for routing protocol.

Parameter	Value	Parameter	Value
Scene size	100 m × 100 m	Mobility model	Random way out
Moving speed	0–50 m/s	Number of nodes in network	40
Initial energy of node	100 J	Packet rate β	1–10 packets/s
MAC protocol	IEEE 802.11	Data rate	1 Mbps
Communication range	250 m	Packet size	512 Bytes
Pheromone heuristic factor α	0.7	Expected heuristic factor β	0.3
Initial pheromone $\tau_{0}$	10	Pheromone evaporate rate	0.7
Simulation time	200 s	Evaporation duration	1 s
Number of iterations	100	Ant number in each iteration	10

## References

[1] Hyland M., Mullins B.E., Baldwin R.O., Temple M.A. Simulation-based performance evaluation of mobile ad hoc routing protocols in a swarm of unmanned aerial vehicles. Proceedings of the 21st International Conference on Advanced Information Networking and Applications Workshops (AINAW’07).

[2] Zhang H., Wang X., Memarmoshrefi P., Hogrefe D. (2017). A Survey of Ant Colony Optimization Based Routing Protocols for Mobile Ad Hoc Networks. IEEE Access.

[3] Sun W., Yang Z., Zhang X., Liu Y. (2014). Energy-efficient neighbor discovery in mobile ad hoc and wireless sensor networks: A survey. IEEE Commun. Surv. Tutor..

[4] Polastre J., Szewczyk R., Mainwaring A., Culler D., Anderson J. (2004). Analysis of wireless sensor networks for habitat monitoring. Wireless Sensor Networks.

[5] Liu Y., Mao X., He Y., Liu K., Gong W., Wang J. (2013). CitySee: Not only a wireless sensor network. IEEE Netw..

[6] Yang Z., Li M., Liu Y. Sea depth measurement with restricted floating sensors. Proceedings of the 28th IEEE International Real-Time Systems Symposium (RTSS 2007).

[7] Huang J.H., Amjad S., Mishra S. (2005). Cenwits: A sensor-based loosely coupled search and rescue system using witnesses. Proceedings of the 3rd International Conference on Embedded Networked Sensor Systems.

[8] Kusý B., Amundson I., Sallai J., Völgyesi P., Lédeczi A., Koutsoukos X. (2010). RF Doppler Shift-based Mobile Sensor Tracking and Navigation. ACM Trans. Sen. Netw..

[9] Miluzzo E., Lane N.D., Fodor K., Peterson R., Lu H., Musolesi M., Eisenman S.B., Zheng X., Campbell A.T. (2008). Sensing meets mobile social networks: The design, implementation and evaluation of the cenceme application. Proceedings of the 6th ACM Conference on Embedded Network Sensor Systems.

[10] Tseng Y.C., Hsu C.S., Hsieh T.Y. (2003). Power-saving protocols for IEEE 802.11-based multi-hop ad hoc networks. Comput. Netw..

[11] Doshi S., Bhandare S., Brown T.X. (2002). An on-demand minimum energy routing protocol for a wireless ad hoc network. ACM SIGMOBILE Mob. Comput. Commun. Rev..

[12] Misra A., Banerjee S. MRPC: Maximizing network lifetime for reliable routing in wireless environments. Proceedings of the 2002 IEEE Wireless Communications and Networking Conference (WCNC2002).

[13] Dressler F., Akan O.B. (2010). A survey on bio-inspired networking. Comput. Netw..

[14] Bandgar A.R., Thorat S.A. An improved location-aware ant colony optimization based routing algorithm for MANETs. Proceedings of the 2013 Fourth International Conference on Computing, Communications and Networking Technologies (ICCCNT).

[15] Yu Y., Li Y., Li J. (2015). Nonparametric modeling of magnetorheological elastomer base isolator based on artificial neural network optimized by ant colony algorithm. J. Intell. Mater. Syst. Struct..

[16] Di Caro G., Dorigo M. (1998). AntNet: Distributed stigmergetic control for communications networks. J. Artif. Intell. Res..

[17] Baras J.S., Mehta H. A Probabilistic Emergent Routing Algorithm for Mobile Ad Hoc Networks. Proceedings of the Modeling and Optimization in Mobile, Ad Hoc and Wireless Networks (WiOpt’03).

[18] Osagie E., Thulasiraman P., Thulasiram R.K. PACONET: ImProved ant colony optimization routing algorithm for mobile ad hoc networks. Proceedings of the 22nd International Conference on Advanced Information Networking and Applications (AINA 2008).

[19] Bullnheimer B., Hartl R., Strauss C. (1999). A New Rank Based Version of the Ant System—A Computational Study.

[20] Woungang I., Dhurandher S.K., Obaidat M.S., Ferworn A., Shah W. An ant-swarm inspired energy-efficient ad hoc on-demand routing protocol for mobile ad hoc networks. Proceedings of the 2013 IEEE International Conference on Communications (ICC).

[21] de Figueiredo Marques V., Kniess J., Parpinelli R.S. An Energy Efficient Mesh LNN Routing Protocol Based on Ant Colony optimization. Proceedings of the 2018 IEEE 16th International Conference on Industrial Informatics (INDIN).

[22] Misra S., Dhurandher S.K., Obaidat M.S., Gupta P., Verma K., Narula P. (2010). An ant swarm-inspired energy-aware routing protocol for wireless ad-hoc networks. J. Syst. Softw..

[23] Zhou J., Wang X., Tan H., Deng Y. (2015). Ant Colony-Based Energy Control Routing Protocol for Mobile Ad Hoc Networks. Proceedings of the International Conference on Wireless Algorithms, Systems, and Applications.

[24] Prabaharan S.B., Ponnusamy R. Secure and energy efficient MANET routing incorporating trust values using hybrid ACO. Proceedings of the 2016 International Conference on Computer Communication and Informatics (ICCCI).

[25] Nath S., Banik S., Seal A., Sarkar S.K. Optimizing MANET routing in AODV: An hybridization approach of ACO and firefly algorithm. Proceedings of the 2016 Second International Conference on Research in Computational Intelligence and Communication Networks (ICRCICN).

[26] Qu W., Wang X. (2017). An Energy-Saving Routing Strategy Based on Ant Colony Optimization in Wireless Sensor Networks. Proceedings of the International Conference in Swarm Intelligence.

[27] Gaillard G., Barthel D., Theoleyre F., Valois F. (2016). Kausa: KPI-aware Scheduling Algorithm for Multi-flow in Multi-hop IoT Networks. Proceedings of the International Conference on Ad-Hoc Networks and Wireless.

[28] Dorigo M. (1992). Optimization, Learning and Natural Algorithms. Ph.D. Thesis.

[29] Heinzelman W.B., Chandrakasan A.P., Balakrishnan H. (2002). An application-specific protocol architecture for wireless microsensor networks. IEEE Trans. Wirel. Commun..

[30] Vijayalakshmi P., Francis S.A.J., Dinakaran J.A. (2016). A robust energy efficient ant colony optimization routing algorithm for multi-hop ad hoc networks in MANETs. Wirel. Netw..

[31] Kim D., Garcia-Luna-Aceves J., Obraczka K., Cano J.C., Manzoni P. Power-aware routing based on the energy drain rate for mobile ad hoc networks. Proceedings of the 2002 Eleventh International Conference on Computer Communications and Networks.

[32] Haibin D. (2005). Ant Colony Algorithm: Theory and Applications.

[33] Stutzle T., Hoos H. MAX-MIN ant system and local search for the traveling salesman problem. Proceedings of the IEEE International Conference on Evolutionary Computation.

